# The Prevention of Rheumatic Endocarditis

**Published:** 1904-07-02

**Authors:** 


### HOSPITAL CLINICS.

### THE PREVENTION OF RHEUMATIC ENDOCARDITIS.

In the Harveian oration recently delivered before the Royal College of Physicians, Dr. Richard Caton recurred to a topic which he has on many previous occasions urged on the attention of his professional brethren. The repetition can be justified, if justification be necessary, by two considerations. First is the importance of the subject—the prevention of cardiac valvular disease in rheumatism. And in the second place the orator is doubtless aware of the fact that in many instances effective audience is only granted to those who fail not in the repeated presentation of their theme. The sum of human misery—physical and mental—caused by organic heart disease can neither be imagined or described. It does not, it is true, present itself to public attention in the dramatic fashion of some epidemic outbreak which rapidly decimates an entire population. But, on the other hand, it never fails in its tale of victims, and many of these endure for years both practical disabilities and real and imagined risks which shadow and darken all their days. Not at occasional intervals but with a ceaseless and unrelenting steadiness is this heavy toll demanded of a definite percentage of our fellow creatures. If this dark area of misfortune and suffering can be lessened, even if the diminution be only a fractional one, it is not a small matter, and attention may surely be asked to the voice of a physician who through good report and evil report has maintained that by appropriate methods this end can in some measure be accomplished. Dr. Caton has no particularly novel, and certainly no sensational, gospel to preach. He has quietly and perseveringly pursued his clinical methods and clinical observations, and by this experience he is content to abide. In an age which demands demonstration and proof this will not appeal to minds of a certain order, but to those who recognise the practical ends of the medical art, and the necessary limitations which attend it, we may hope that Dr. Caton will not speak in vain.

The principles of his method are simple enough. They include the prolonged adoption of complete rest so as to reduce the chance of any rheumatic attack producing disease of the endocardium covering the cardiac valves; the use of sodium or potassium iodide to lower arterial tension and to promote the absorption of morbid exudations; and mild counter-irritation over the course of the upper four dorsal nerves, so as to influence reflexly the cardiac vaso-motor and trophic nerves and ganglia. These, it will be admitted, are reasonable and proper measures to adopt, and it is to be noted that the physician who has used them for 20 years is convinced of their ability to diminish in a most material degree the frequency of chronic valvular disease of the heart. Here, again, actual demonstration is, of course, impossible; and it is to be feared that partly from this cause, partly from a widespread therapeutic scepticism, the methods proposed are either no adopted at all, or are put into action in an incomplete and half-hearted fashion. Yet to prevent valvular disease of the heart would be a triumph, a "relief of man's estate," hardly less valuable than ambitions which are now commanding some of the best energies of the profession.

Unfortunately, for some reason or other, cardiac valve disease seems to be accepted as an inevitable complication in a certain proportion of cases of rheumatic fever. Against such a fatalistic doctrine it were well to maintain an energetic protest, and for this reason, if for no other, we consider Dr. Caton has deserved well of his generation. Another suggestion having a similar object to the one he advances, has also met with scanty recognition, yet it may claim a thoroughly reasonable foundation. That suggestion arises from the consideration of some few elementary facts in the natural history of rheumatic disease. These may be briefly stated as follows: Rheumatism in childhood occurs in much more subtle and insidious forms than it does in adults; these forms are all at least as liable to be complicated by endocarditis as is the acute polyarthritis of older patients; against such a contingency the best protection is early and complete rest; such rest is often not secured because parents with a rheumatic strain are quite unaware of the serious nature and grave risks of what they regard as the minor and trivial ailments of their children. From these facts it is argued that the best way to obtain for children in rheumatic families the protective influence of rest is to forewarn the parents on the matter. An opportunity to do this presents itself whenever the medical attendant becomes aware in the course of his professional experience that he is dealing with those whose tissue proclivities include a tendency to rheumatic disease. His duty, in such circumstances, it is suggested, is not limited to the care and treatment of the individual patient. On the contrary, it includes all other members of the family and more especially those who, in consequence of their age, are liable to forms of rheumatism that do not compel the prompt and complete rest which is necessarily entailed by an acute polyarthritis. Every practitioner sees cases, and these not few in number, where a cardiac lesion has occurred in early life and without any incident recognised as rheumatic by the parents. The contention now is that were parents informed in advance of the special risks and perils of rheumatism in childhood many of these cases would be avoided, so far at least as the cardiac valve disease is concerned. And, in any event, it is the duty of the profession to take this step, seeing that, according to the best knowledge, it is to early and complete rest that we must mainly look as a prophylactic measure against endocarditis and allied lesions in rheumatic disease. Dr. Caton's measures are sound and wise, and are certainly to be urged upon the profession as good practice when a rheumatic attack is in progress. But they do not touch that large proportion of rheumatic cases which receive such names as tonsillitis, chorea, pleurisy, growing pains, etc., etc. Many instances of these never come under the care of a medical man, and are never suspected by the parents as entailing such a grave risk as organic heart disease. The proposal to which allusion is now made covers many of these, and by intervening at the very earliest moment promises to diminish, perhaps considerably, the number of those whose lives are burdened and shortened by a cardiac lesion which took origin in an illness that was never considered to demand either medical treatment or even much domestic care. Protection against this neglect can only be secured when each individual member of the profession realises that the discovery of rheumatism demands not only the cure of the patient but the protection of his relatives.

